# Combining TRAIL with PI3 Kinase or HSP90 inhibitors enhances apoptosis in colorectal cancer cells via suppression of survival signaling

**DOI:** 10.18632/oncotarget.1162

**Published:** 2013-07-14

**Authors:** Grazia Saturno, Melanie Valenti, Alexis De Haven Brandon, George V. Thomas, Suzanne Eccles, Paul A. Clarke, Paul Workman

**Affiliations:** ^1^ Cancer Research UK Cancer Therapeutics Unit, Division of Cancer Therapeutics, The Institute of Cancer Research, London, UK.; ^2^ Divisions of Cancer Biology and Clinical Studies, The Institute of Cancer Research, London, UK.; ^3^ Present address: Molecular Oncology Team, Paterson Institute for Cancer Research, The University of Manchester, Wilmslow Road, Manchester UK; ^4^ Present address: HSU Knight Cancer Institute, Oregon Health and Science University, Portland, OR, USA.

**Keywords:** TRAIL, PI3 Kinase/mTOR, HSP90, apoptosis, colorectal cancer

## Abstract

TRAIL has been shown to induce apoptosis in cancer cells, but in some cases they fail to respond to this ligand. We explored the ability of representative phosphatidylinositol-3-kinase (PI3 Kinase)/mTOR and HSP90 inhibitors to overcome TRAIL resistance by increasing apoptosis in colorectal cancer models. We determined the sensitivity of 27 human colorectal cancer and 2 non-transformed colon epithelial cell lines to TRAIL treatment. A subset of the cancer cell lines with a range of responses to TRAIL was selected from the panel for treatment with TRAIL combined with the PI3 Kinase/mTOR inhibitor PI-103 or the HSP90 inhibitor 17-AAG (tanespimycin). Two TRAIL-resistant cell lines were selected for *in vivo* combination studies with TRAIL and 17-AAG. We found that 13 colorectal cancer cell lines and the 2 non-transformed colon epithelial cell lines were resistant to TRAIL. We demonstrated that co-treatment of TRAIL and PI-103 or 17-AAG was synergistic or additive and significantly enhanced apoptosis in colorectal cancer cells. This was associated with decreased expression or activity of survival protein biomarkers such as ERBB2, AKT, IKKα and XIAP. In contrast, the effect of the combination treatments in non-transformed colon cells was minimal. We show here for the first time that co-treatment *in vivo* with TRAIL and 17-AAG in two TRAIL-resistant human colorectal cancer xenograft models resulted in significantly greater tumor growth inhibition compared to single treatments. We propose that combining TRAIL with PI3 Kinase/mTOR or HSP90 inhibitors has therapeutic potential in the treatment of TRAIL-resistant colorectal cancers.

## INTRODUCTION

Colorectal cancer is the third most common cancer in UK and the second leading cause of cancer-related death in US, with around 40,000 and 140,000 new cases registered each year respectively [1,2]. The current standard treatment for patients with colorectal cancer is surgical resection followed by adjuvant chemotherapy for those patients who can tolerate the chemotherapy regimen [3]. In addition to cytotoxic chemotherapy, some agents targeted against specific molecular pathways have been evaluated in colorectal cancer and it has been demonstrated that treatment with these drugs alone or in combination with standard chemotherapy can result in a significant survival advantage, e.g. cetuximab as a monotherapy or in combination with irinotecan or oxaliplatin [3-5].

Apoptosis is a tightly regulated process through which cells are programmed to die: there are two types of apoptosis, regulated by the intrinsic and the extrinsic pathways [6]. TRAIL (TNF related apoptosis inducing ligand) induces apoptosis through an extrinsic pathway by binding to the death receptors DR4 and DR5. The ligand-bound receptors interact with the adaptor protein FADD (Fas (TNFRSF6)-associated via death domain) and caspase 8 forming the DISC (Death–Inducing Signalling Complex) that directly activates terminal caspases such as caspase 3 [7]. At this stage caspase 8 cleavage and activation can be inhibited by the recruitment of FLIP (FLICE-inhibitory protein) within the DISC [8-10].

It has been demonstrated that TRAIL can specifically target tumor cells, inducing apoptosis without affecting normal tissues that are generally resistant to the ligand [11]. Recombinant TRAIL or high-affinity agonist monoclonal antibodies against TRAIL death-receptors are now in Phase I/II clinical trials [12-15]. However, despite the potential therapeutic specificity for cancer cells, intrinsic resistance to TRAIL-induced apoptosis has been identified as a current challenge [15,16]. This TRAIL resistance can be due to downstream molecules involved in apoptosis such as FLIP, BAD or BAX [8,17]. Finally, TRAIL can also activate survival pathways regulated by PI3 Kinase and NFκ-B leading to a pro-survival effect via a mechanism that is not yet fully defined [18,19]. Therefore, the use of therapies that combine TRAIL or TRAIL receptor agonist antibodies together with drugs that target potential mechanisms of TRAIL-resistance represents a very attractive strategy for cancer treatment.

PI3 Kinase and HSP90 are key proteins and drug targets with a major role in the control of cell survival, cell growth and apoptosis by modulating the activity of a number of pathways [20-27]. The PI3 Kinase pathway is frequently deregulated in cancer and signaling downstream of PI3 Kinase involves proteins such as AKT, PDK and mTOR that control multiple cellular mechanisms [21, 22]. HSP90 is a chaperone protein responsible for the correct folding, stability and activation of client proteins. It plays a fundamental cellular role in normal and stress conditions as well as in pathological states such as cancer [24-27]. Inhibiting HSP90 leads to the loss of activity and degradation of client proteins, among which are key components of the PI3 Kinase signaling pathway that include ERBB2, AKT and IKKα [24-28]. IKK (Inhibitor κB Kinase) is the major activator of NFκ-B which is responsible for transcription of c-IAPs and XIAP (Inhibitors of Apoptosis) that directly block caspase activation [29].

Stimulated by the important roles of PI3 Kinase and HSP90 chaperone pathways in cancer and the potential for selectivity based on the dependence of cancer cells on these pathways, inhibitors of both pathways have been identified and are now undergoing clinical trials [21-27]. Several HSP90 inhibitors have been developed; among these is the potent derivative of the natural product geldanamycin 17-AAG (tanespimycin) which was the first HSP90 inhibitor to enter clinical trials [24-27]. It has been demonstrated previously that inhibition of HSP90 by geldanamycin or 17-AAG can sensitize some cancer cells to TRAIL-induced apoptosis *in vitro* [30-32]. PI-103 is a prototype PI3 Kinase inhibitor that potently and selectively targets class I PI3 Kinases and mTOR [33,34]. Previous studies have suggested that signaling through PI3 Kinase can prevent TRAIL-induced apoptosis in different cancer cell types [35,36]; however, these studies were limited to using LY294002, an early PI3 Kinase inhibitor that has weak potency and off-target activity on protein kinases such as casein kinase 2 [37]. It has been reported that PI-103 increases the effect of TRAIL in glioma [38] and neuroblastoma models [39]. Based on these data, we hypothesized that inhibitors of PI3 Kinase/mTOR or HSP90 could enhance sensitivity to TRAIL in TRAIL-resistant colorectal cancer cells by modulating survival signaling.

Here, our aims were to explore the ability of representative, specific PI3 Kinase/mTOR or HSP90 inhibitors to reverse resistance to TRAIL-induced apoptosis in human colorectal cancer. We demonstrate that combinations of TRAIL and PI-103 or 17-AAG were synergistic or additive and induced increased apoptosis in TRAIL-resistant human colorectal cancer cells with the simultaneous inhibition of the activity or expression of ERBB2, AKT, IKKα and XIAP. In contrast, this effect was minimal in non-transformed CO841 human colon epithelial cells, indicating the potential for differential therapeutic selectivity. We also demonstrate here, to our knowledge for the first time, the promising *in vivo* efficacy of combinatorial treatment with TRAIL and 17-AAG in two TRAIL-resistant human colorectal tumor xenograft models. Associated biomarker changes were consistent with the proposed mechanism of reduced survival signaling. Our results indicate the therapeutic potential of combinatorial therapy with PI3 Kinase/mTOR or HSP90 inhibitors in colorectal cancer and suggest useful mechanism-based pharmacodynamic biomarkers.

## RESULTS

### TRAIL SENSITIVITY IN A PANEL OF HUMAN COLORECTAL CANCER AND NON-TRANSFORMED CELL LINES

**Figure 1:**
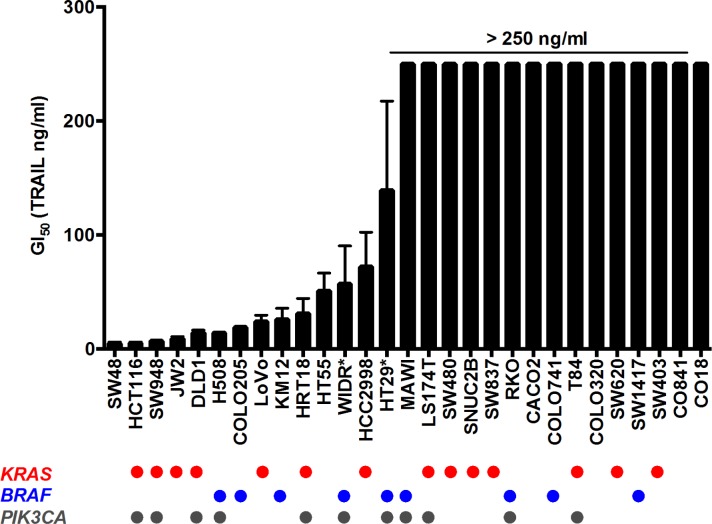
TRAIL sensitivity and mutation status of human colorectal and non-transformed cell line panel Cells were treated for 96 h with TRAIL and cell number was measured by SRB; GI_50_ for each cell line is represented on the Y axis, >250 ng/ml indicates that the GI_50_ was not achieved below this concentration. N=3, error bars are standard deviations; (*) denotes N=4 for WIDR and HT29 cell lines. CO841 and CO18 are non-transformed human colon epithelial cell lines while the remaining are cancer lines. Mutation status for *KRAS*, *BRAF* and *PIK3CA* are reported in the lower panel, each circle representing the presence of the mutated form of the protein for each cell line.

A panel of 27 human colorectal cancer and 2 non-transformed human colon epithelial cell lines were screened for TRAIL sensitivity by determining GI_50_ values at 96h using the SRB assay. Of the 29 lines, 14 responded to TRAIL treatment with GI_50_ values ranging from 4.6 to 139 ng/ml. A GI_50_ could not be determined for the remaining resistant cells even at the highest concentration of 250 ng/ml TRAIL (Fig. 1). TRAIL sensitivity was not related to the presence of activating oncogenic *KRAS*, *BRAF* and *PIK3CA* mutations common to colorectal cancer (Fig. 1).

**Figure 2:**
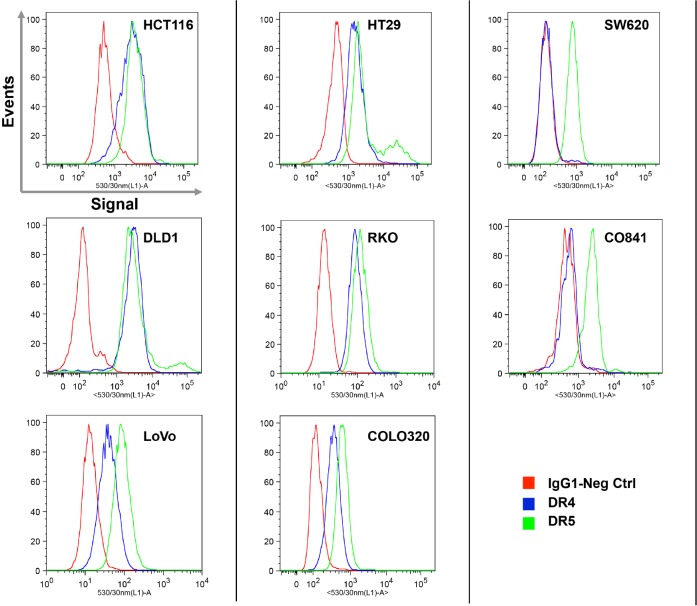
Expression analysis of TRAIL receptors DR4 and DR5 measured by flow cytometry Histograms represent the IgG1 negative control (red), DR4 (blue) and DR5 (green) on the surface of live cell populations. Cells were counterstained with propidium iodide (PI) and PI-positive dead cells were excluded from analysis. The intensity of the fluorescent signal is reported on the X axis. Plots are representative of three independent experiments. Cells were human colorectal cancers, as indicated, apart from the CO841 non-transformed human epithelial cell line.

The interaction between TRAIL and its receptors is the first step triggering apoptosis and TRAIL sensitivity may be influenced by the level of expression of these receptors on the cell surface [40]. Therefore, DR4 and DR5 TRAIL receptor expression was analyzed by flow cytometry in a subset of 7 colorectal cancer lines and the non-transformed CO841 cells. There was no correlation between expression of TRAIL receptors DR4 and DR5 on the cell surface and resistance to TRAIL (Fig. 2). With one exception, all tumor cell lines analyzed exhibited cell surface expression of DR4 and DR5, irrespective of sensitivity to TRAIL. The exception was the SW620 tumor cell line that expressed DR5, but not DR4, a pattern similar to the non-transformed CO841 cells (Fig. 2). HCT116, LoVo (both sensitive) and RKO (resistant) tumor cells were also analyzed for the expression of decoy receptors DcR1 and DcR2. All the three lines expressed detectable DcR2 and lower levels of DcR1 (Supplementary Fig. 1).

FLIP (FLICE-inhibitory protein), a negative regulator of TRAIL-induced apoptosis, is considered as one of the key proteins responsible for TRAIL resistance [41]. Cells were therefore analyzed for FLIP protein expression by immunoblot, but no differences were found among a selection of 7 tumor cell lines with different degrees of sensitivity to TRAIL (Supplemetary Fig. 2A). In addition, inhibition of FLIP expression by siRNA in one of these tumor cell lines, RKO, did not reverse the resistance to TRAIL, as assessed by cell number or PARP cleavage (Supplementary Fig. 2B-E). This suggested that elevated basal expression of FLIP protein in the TRAIL-resistant cells compared to the TRAIL-sensitive cells was unlikely to be a cause of resistance, at least for the 5 TRAIL-resistant lines tested here.

### TIME-DEPENDENCE OF TRAIL RESPONSE

HCT116 (sensitive, GI_50_ = 5 ng/ml), HT29 (less sensitive, GI_50_ = 139 ng/ml), RKO (resistant, GI_50_ > 250 ng/ml), SW620 (resistant, GI_50_ > 250 ng/ml) colorectal cancer and CO841 normal colon epithelium (resistant, GI_50_ > 250 ng/ml) cell lines were selected for further analysis as representative models to explore the mechanism of TRAIL resistance in colon cancer.

**Figure 3:**
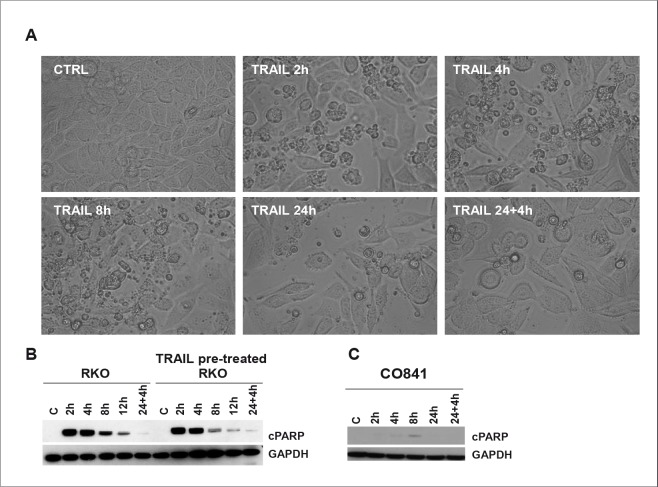
TRAIL response time course in RKO colorectal cancer and CO841 non-transformed colon epithelial cells Panel A: images of RKO cells at time 0 or treated with TRAIL 200 ng/ml for 2, 4, 8, 24 h or 24 h plus additional 4 h with fresh 200 ng/ml TRAIL were taken using an inverted light microscope (Leica), 20X magnification. Some rounded and small apoptotic cells were detected at 2, 4 and 8h. Cleavage of PARP was assessed by immunoblot at time 0 (C), 2, 4, 8, 12 and 24 plus additional 4 h 200 ng/ml TRAIL treatment (panel B, left). Panel B, right, shows the effects of the same treatment in TRAIL-pretreated RKO cancer cells after 4 days of recovery. Panel C shows results for a similar time course in non-transformed CO841 colon epithelial cells.

During TRAIL treatment we observed that resistant tumor cell lines, such as RKO, responded to the apoptotic stimulus at early stages of TRAIL treatment, but at later times of exposure (24h and later) the apoptotic response defined by PARP cleavage was lost, despite continued exposure to TRAIL (Fig. 3).

TRAIL-sensitive HCT116 tumor cells were analyzed after TRAIL treatment (5 ng/ml) and images were taken at 2, 4, 8 and 24h post treatment. Apoptosis was observed as early as 2h post-treatment and by 24h essentially all cells had died; cleavage of PARP continued to increase throughout the time-course up to 24h (Supplementary Fig. 3). Surprisingly, apoptosis was observed at 2, 4 and 8h following treatment of resistant RKO tumor cells with a high concentration of TRAIL (200 ng/ml) (Fig. 3A); however, at 24h, even after the addition of fresh TRAIL, cells showed far less apoptosis. Immunoblotting for cleaved PARP content confirmed the induction of apoptosis at 2-12h (Fig. 3 B, left), but at 24h, again even after adding fresh TRAIL, the cells no longer exhibited PARP cleavage.

Next, we tested the potential persistence of the acquired loss of apoptosis. After 24h exposure to TRAIL, RKO cells were washed and left in TRAIL-free culture media for 4 days before retreatment with TRAIL following the same time course described above. The cells exhibited the same apoptotic response to the high concentration of TRAIL for up to 8-12h, but as before showed no cleavage of PARP at 24h, even when fresh TRAIL was added for a further 4h (Fig. 3B, right). Song and colleagues have reported a similar effect in human TRAIL-resistant prostate adenocarcinoma cells [17].

Our data demonstrated that TRAIL-resistant RKO colorectal cancer cells were able to activate the apoptotic pathway after TRAIL treatment, albeit at high concentrations, but became insensitive beyond 12h exposure to the ligand. We observed the same effect in other TRAIL-resistant colorectal cancer cell lines such as HT29 and COLO320 (data not shown). Importantly, the PARP cleavage observed in RKO and HCT116 tumor cells was not observed in non-transformed CO841 colon epithelial cells, suggesting that the non-tumorigenic cells have a mechanism of resistance to TRAIL that is likely to be different from the TRAIL-resistant colorectal cancer cell lines (Fig. 3C and Supplementary Fig. 4).

### OVERCOMING TRAIL RESISTANCE WITH PI-103 OR 17-AAG

Colorectal cancer cells were treated with TRAIL alone or in combination with the representative PI3 Kinase/mTOR inhibitor, PI-103 or the representative HSP90 inhibitor, 17-AAG. HT29 cells are resistant to PI-103, most likely through rapid glucuronidation to an inactive metabolite [33], and so were treated with an alternative, more metabolically stable PI3 Kinase inhibitor GDC-0941 that is currently undergoing clinical trial [42,43].

**Figure 4:**
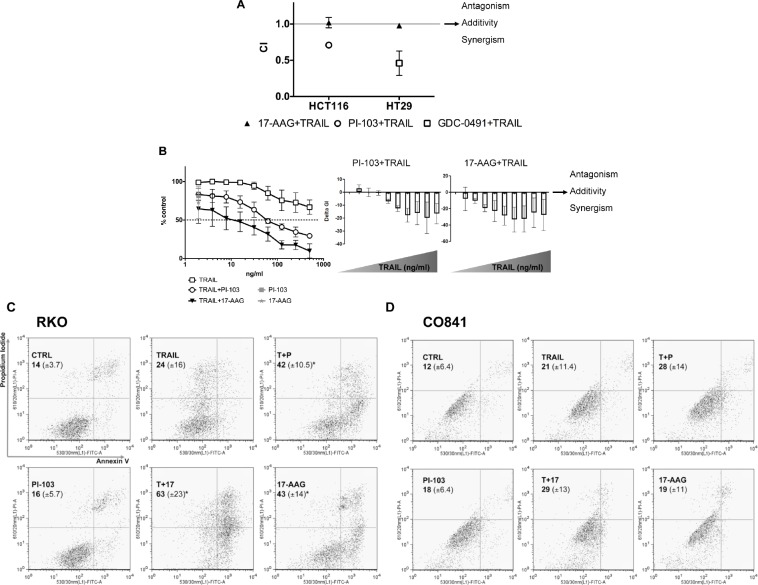
Combination treatments in colorectal cancer cells and non-transformed colon epithelial cells HCT116 and HT29 cancer cells were treated with 17-AAG (GI_50_ = 47 and 11 nM respectively) and TRAIL (GI_50_ = 5 and 139 ng/ml respectively), or PI-103 (GI_50_ = 1 μM) and TRAIL or GDC-0491 (GI_50_ = 1.367 μM) and TRAIL for 96 h and Combination Indices are shown in panel A: CI<1 synergism, CI=1 additivity and CI>1 antagonism. N=3, bars are standard errors. Panel B: TRAIL treatment of RKO cancer cells (from 1.95 ng/ml up to 500 ng/ml) with or without a fixed dose of PI-103 (100 nM) or 17-AAG (33.5 nM). Additive or synergistic effects are shown on the right: values above zero indicate antagonism, those equal to zero indicate additivity and those below zero indicate synergism. N=3, error bars are standard deviations. Panels C and D: apoptosis was measured in both RKO colorectal cancer and non-transformed CO841 colon cells by flow cytometry using FITC-Annexin V and PI staining. Percentage means (± s.d.) of FITC-Annexin V plus FITC/PI stained cells are reported for each treatment, (*) denotes p≤0.05. Plots are representative of three independent experiments.

Combination treatments were performed in TRAIL-sensitive HCT116 and TRAIL-intermediately sensitive HT29 colorectal cancer cell lines. CIs (Combination index) were calculated in HCT116 cells for PI-103 or 17-AAG plus TRAIL and in HT29 cells for GDC-0941 or 17-AAG plus TRAIL. The analysis demonstrated synergism in both HCT116 and HT29 tumor cells for the combination of PI3 Kinase inhibitors and TRAIL, while additivity was observed in both cell lines for 17-AAG in combination with TRAIL (Fig. 4A). Importantly, in TRAIL-resistant RKO tumor cells, the addition of either of the inhibitors, but particularly 17-AAG, markedly sensitized the response to TRAIL in a concentration-dependent manner, with both co-treatments exhibiting a synergistic response (Fig. 4B).

Apoptosis quantification was carried out in RKO, HCT116 and CO841 cells by flow cytometry analysis using FITC-Annexin V after treatment at 2.5 × GI_50_ PI-103 or 17-AAG and 200 ng/ml TRAIL. Significantly increased apoptosis (p<0.05) was detected in TRAIL-resistant RKO colorectal cancer cells treated for 24h with TRAIL plus PI-103 or 17-AAG as compared to single treatments (Fig. 4C). Enhanced apoptosis was also seen in TRAIL-sensitive HCT116 colorectal tumor cells, although in these cells TRAIL alone at the concentration of 2.5×GI_50_ induced apoptosis in more than 50% of the cells (Supplementary Fig. 5). In contrast to the cancer cell lines, the non-transformed TRAIL-resistant CO841 colon epithelial cells failed to show a significant increase in apoptosis for either single or combination treatments (Fig. 4D). These results support the proposed hypothesis that the synergistic responses seen in colorectal cancer cells with the combination treatments are associated with increased apoptosis, and indicate a potential for therapeutic selectivity between cancer and normal cells.

### COMBINATION TREATMENT CAUSES INCREASED CASPASE CLEAVAGE AND DECREASED XIAP EXPRESSION

**Figure 5:**
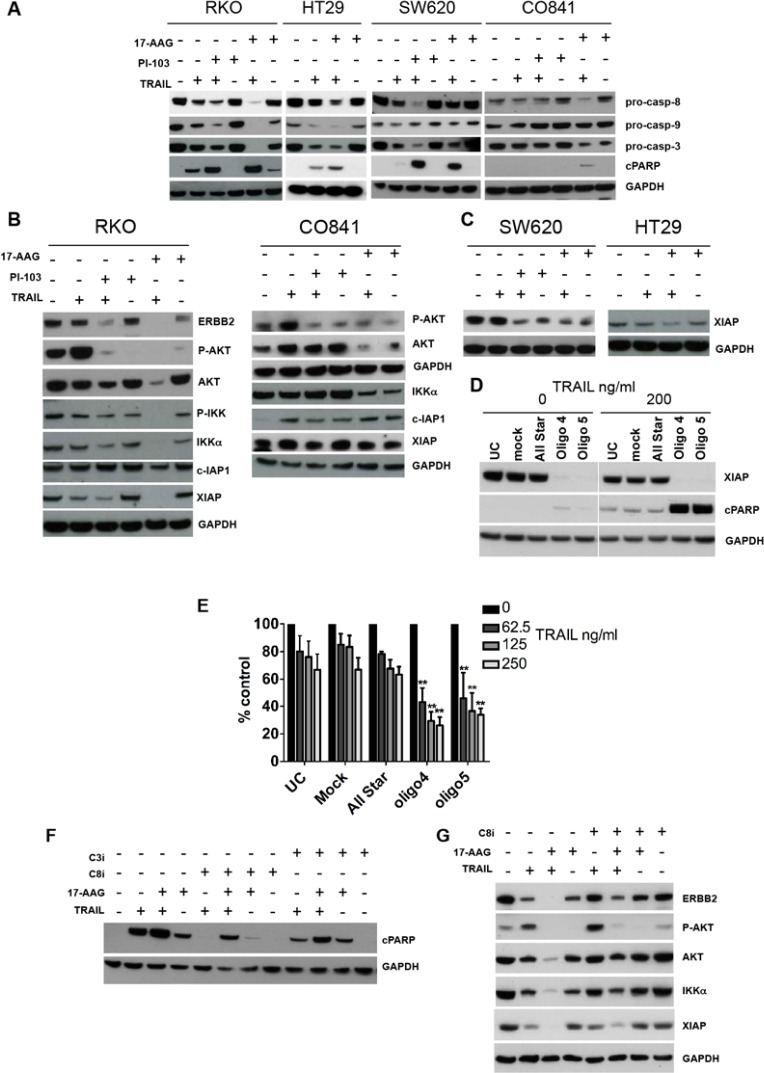
Effect of combination treatments on the molecular signature of apoptosis and survival pathways in TRAIL-resistant colorectal cancer and non-transformed colon epithelial cells HT29 cancer cells were treated with 27.5 nM 17-AAG and 347.5 ng/ml TRAIL; RKO, SW620 cancer and CO841 non-transformed colon cells were treated with 167.5-500 nM 17-AAG, 500-1000 nM PI-103 and 200 ng/ml TRAIL. Panel A: immunoblots for pro-caspase 8, 9, 3 levels and PARP cleavage after single or combination treatments are shown. A decrease in pro-caspase levels indicates greater caspase cleavage. An increase in cleaved PARP levels indicates greater apoptosis. Panel B: immunoblot for ERBB2, AKT and AKT^Ser473^, IKKα, IKKα^Ser176^, c-IAP1 and XIAP in TRAIL-resistant RKO cells and CO841 non-transformed CO841 cells; IKKα^Ser176^ and ERBB2 were not detectable in these cells (data not shown). Immunoblot analysis for XIAP is shown in panel C, for TRAIL less sensitive HT29 and TRAIL-resistant SW620. Panel D, left: effects of XIAP siRNA silencing in RKO cancer cells, UC=untreated control, Mock = transfection lipid, All Star = oligo control. Cells were transfected for 48 h and treated for 24 h with TRAIL 200 ng/ml. XIAP and cleaved PARP levels were assessed by immunoblot. Panel E shows results of the SRB antiproliferative assay: cells were transfected for 48 h and then treated with three different concentrations of TRAIL for 72 h. N=3, error bars are standard deviations, (*) denotes p < 0.05, (**) denotes p < 0.01. RKO cells were pre-treated with 20 μM Z-IETD-FMK (Panel F and G) or Z-DEVD-FMK (Panel F) for 1 h followed by 4 h of 167 nM 17-AAG alone or in combination with 200 ng/ml TRAIL for further 24 h. Panel F: immunoblot analysis for the cleavage of PARP. Panel G: immunoblot analysis for IKKα, XIAP, ERBB2, AKT and AKT^Ser473^.

Next, we investigated biomarker changes underlying the observed increase in apoptosis following the combination treatment in a range of TRAIL-resistant colorectal cancer and the non-transformed colon cell lines. Intermediately sensitive HT29 tumor cells, resistant RKO and SW620 tumor cell lines and non-transformed CO841 cells were treated for 24h with 17-AAG or PI-103 plus TRAIL. In addition to determining PARP cleavage following caspase activation, we also measured the decrease in pro-caspase 3, 8 and 9 levels that indicates activation of the proteolytoic caspase cascade. Co-treatment with TRAIL plus 17-AAG or PI-103 generally induced a decrease in the levels of pro-caspases compared to individual treatments in HT29 tumor cells and the TRAIL-resistant RKO and SW620 colorectal cancer cells (Fig. 5A) as well as HCT116 TRAIL-sensitive cells (Supplementary Fig. 6). In non-transformed TRAIL-resistant CO841 colon cells, only weak reduction of pro-caspase 8 was observed and only for the co-treatment with 17-AAG (Fig. 5A). The non-transformed CO841 cells also showed no or weak cleavage of caspase 3 when treated with 17-AAG or 17-AAG and TRAIL.

In association with decreased pro-caspase levels, we observed an increase in the cleaved form of PARP following co-treatment of colorectal cancer cells with 17-AAG or PI-103 and TRAIL as compared to single agent treatments (Fig. 5A and Supplementary Fig. 6). In contrast, much weaker PARP cleavage was seen in non-transformed CO841 cells co-treated with 17-AAG and TRAIL.

Since a significant increase in colorectal cancer cell apoptosis was observed after combination treatments, we further investigated the status of molecules involved in regulating the apoptotic program and surviving/cell growth pathways. We focused on IKKα, c-IAP1 and XIAP, ERBB2 and AKT as they are key regulators of cell survival that are mechanistically linked to the PI3 Kinase and HSP90 pathways. Both PI-103 and 17-AAG when combined with TRAIL caused a decrease in the levels of IKKα and its phosphorylated form in TRAIL-resistant RKO cancer cells; expression of the caspase inhibitor protein XIAP was also decreased following both co-treatments (Fig. 5B). In addition, the 17-AAG plus TRAIL co-treatment resulted in a modest decrease in levels of the caspase inhibitor c-IAP1.

PI-103 and 17-AAG both decreased AKT^Ser473^ phosphorylation when used as single agents in RKO tumor cells; moreover, when combined with TRAIL they prevented the increase in phospho-AKT caused by the ligand alone (Fig. 5B). This effect of TRAIL on AKT phosphorylation status was also observed in the majority of colorectal cancer cell lines studied, regardless of their sensitivity to TRAIL (data not shown). The non-transformed CO841 colon cells likewise showed the expected decrease in total and phospho-AKT levels following treatment with 17-AAG alone or in combination with TRAIL. IKKα was also depleted in the non-transformed CO841 cells, but not to the same extent as in the TRAIL-resistant RKO or TRAIL-sensitive HCT116 cancer cells (Fig. 5B and Supplementary Fig. 6). Furthermore, in contrast to the results in cancer cells, all the single agent and combination treatments increased c-IAP1 expression in these cells (Fig. 5B). XIAP levels were not altered in non-transformed CO841 colon cells after co-treatment compared to single agents and untreated controls (Fig. 5B). Overall, the non-transformed CO841 colon epithelial cells showed lower modulation of survival proteins in response to the drug combinations when compared to the colorectal cancer lines, indicating that these non-transformed cells are less sensitive to the amplification of the TRAIL apoptotic stimulus induced by co-treatment with PI-103 or 17-AAG. We observed a decrease in XIAP levels following 17-AAG plus TRAIL also in the less TRAIL-sensitive HT29 cancer cells, and in this tumor cell line we noted XIAP basal levels were lower compared to SW620 and RKO cancer cell lines (Fig. 5A and data not shown). In addition, a decrease in XIAP expression following single or combination treatments was observed in SW620 TRAIL-resistant tumor cells (Fig. 5C).

To mimic the effect of combination treatments on XIAP, we transfected the TRAIL-resistant RKO tumor cells with two different XIAP siRNA or control siRNA for 48h and then treated the cells with 200 ng/ml TRAIL for 24h. Silencing XIAP increased apoptosis after TRAIL treatment as shown by a greater level of cleaved PARP (Fig. 5D). XIAP knockdown followed by TRAIL treatment resulted in significantly lower cell viability compared to control siRNA transfected cells (Fig. 5E). This suggests that the synergistic effect of the combination treatments in colorectal cancer cells was consistent with decreased survival signaling resulting in decreased expression of XIAP.

Pre-incubation of RKO cells with 20 μM Z-IETD-FMK, a caspase-8 inhibitor [40], resulted in inhibition of the apoptotic effect induced by TRAIL and TRAIL plus 17-AAG. Pretreatment with the caspase 3 inhibitor Z-DEVD-FMK also decreased PARP cleavage although to a lesser extent than Z-IETD-FMK (Fig. 5F). Furthermore, pretreatment with the Z-IETD-FMK in the combination did not rescue the levels AKT^Ser473^ back to the levels observed when cells were treated with 17-AAG alone (Fig. 5G). XIAP levels were only slightly rescued by caspase 8 inhibition under the same conditions. These observations suggest that the decreases in AKT^Ser473^ and XIAP levels were unlikely to be a direct consequence of caspase 8 activation (Fig. 5G). In contrast, in RKO tumor cells treated with the TRAIL plus 17-AAG combination, Z-IETD-FMK exposure resulted in the levels of depleted HSP90 client proteins such as ERBB2, IKKα and AKT returning to those observed in tumor cells treated with 17-AAG alone (Fig. 5G). This suggested that caspase 8 activation contributed to the potent effects of the TRAIL plus 17-AAG combination on these HSP90 client proteins.

### GROWTH INHIBITION OF TRAIL-RESISTANT COLON TUMOR XENOGRAFTS WITH THE COMBINATION OF TRAIL PLUS 17-AAG

**Figure 6:**
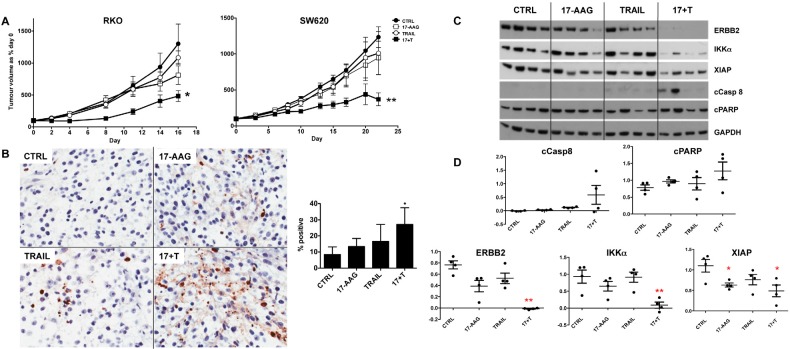
Effectiveness of the 17-AAG plus human recombinant TRAIL combination treatment of established RKO and SW620 human colorectal cancer xenografts in nude mice Panel A: tumor growth volumes are expressed as a percentage of day 0 for untreated control, TRAIL only, 17-AAG only and 17-AAG plus TRAIL. N=8, bars are standard error, (*) denotes p≤0.05 and (**) p ≤0.01 compared to untreated control group. Panel B: paraffin embedded tumor sections from RKO tumors stained with TUNEL (20X magnification) and quantification of positive stained nuclei, (*) denotes p≤0.05 compared to not treated control group; N=4, error bars are standard deviations. Panel C: immunoblot analysis for cleaved caspase 8, cleaved PARP, ERBB2, IKKα and XIAP levels, each lane corresponding to one representative tumor/animal. Panel D: densitometry after normalization to GAPDH loading control, (*) denotes p≤0.05 and (**) p ≤0.01 compared to untreated control group.

To further assess the therapeutic potential of our combinatorial approach, we prioritized the combination of TRAIL plus the representative HSP90 inhibitor 17-AAG. This was based on the generally more pronounced effects of 17-AAG versus PI-103 when combined with TRAIL on IKKα and XIAP, together with the greater suitability of 17-AAG for *in vivo* use [24,34]. We tested the effects of the 17-AAG plus TRAIL combination treatment *in vivo* in two different human colorectal tumor xenograft models, RKO and SW620. Both colorectal cancer cell lines showed resistance to TRAIL as a single agent *in vitro* (Fig. 1) and also *in vivo* as established tumor xenografts (Fig. 6). Importantly, the combination of 17-AAG and TRAIL resulted in greater tumor growth inhibition compared to single agents in both RKO and SW620 colorectal tumor xenografts (Fig. 6A).

Consistent with the effectiveness of the combination treatments, combining 17-AAG and TRAIL significantly increased the percentage of TUNEL-positive apoptotic cells compared to control, whereas only a slight and non-significant increase was observed for the single treatments in the short-term biomarker study (Fig. 6B). Immunoblot analysis showed that cleavage of PARP and activation of caspase 8 was greater in the combination treatment compared to single agents in the same study. Moreover, ERBB2, IKKα levels were significantly lower in the combination treatment group as compared to single agent and control samples; XIAP was significantly lower in 17-AAG and 17-AAG plus TRAIL groups in the short-term study (Fig. 6C, 6D). ERBB2 was identified as the most robust biomarker at the end of treatment; its expression following the long-term therapy experiment shown in Fig. 6A was significantly lower in the SW620 tumors treated with 17-AAG plus TRAIL compared to control or the single agent treatments (Supplementary Fig. 7A). In the RKO tumor model we observed a significant decrease of ERBB2 in all the treatments with p values indicating stronger significance for the combination treatment (Supplementary Fig.7B).

These data show that the combination of 17-AAG plus TRAIL inhibited the growth of colorectal tumor xenografts *in vivo* more effectively than did the single agents via a greater induction of apoptosis and with concomitant biomarker modulation.

## DISCUSSION

The selectivity of TRAIL towards some cancer cells combined with its relatively low toxicity have made it a very attractive potential therapeutic agent in different cancers [7,44]. However, sensitivity to TRAIL may vary between individual tumors, as for example in the case of colorectal cancer demonstrated here and elsewhere [30,41]. Although human recombinant TRAIL as a single agent has been reported to be safe and well tolerated in Phase I clinical trials, only 46% of the patients had stable disease after a second cycle of treatment (>6 months) [44]. This interesting but limited response and lack of activity in the remaining patients may be due to intrinsic or acquired resistance. In view of this, the combination of TRAIL with other therapeutic agents has the potential to improve its efficacy and give a better outcome in a higher percentage of patients.

A number of studies have explored this concept; at present, therapies combined with TRAIL or TRAIL receptor antibodies in clinical trials include cytotoxic regimens including cisplatin or radiotherapy which enhance the intrinsic apoptotic pathway leading to the amplification of TRAIL-induced apoptosis [45]. Combining TRAIL or TRAIL receptor agonists with selective molecularly targeted agents is also being evaluated as a strategy to amplify TRAIL-induced apoptosis [12,13]. The use of targeted therapies in combination with TRAIL has at least two potential advantages: amplification of TRAIL-induced apoptosis and reduction of side-effects compared to combinations involving cytotoxic agents.

The mechanism of resistance to TRAIL is not completely understood. Reports in the literature [18] have variously reported that differential expression of receptors or decoy-receptors, increased expression of anti-apoptotic factors like FLIP or IAPs, or increased activation/expression of oncoproteins involved in cell survival (including ERBB2, PI3 Kinase, AKT and IKK) may all reduce TRAIL sensitivity. Paradoxically, TRAIL itself is also capable of inducing survival pathways such as PI3 Kinase, NFκ-B or MAPK [18]. Activating mutations of oncogenes such as *KRAS*, *BRAF* or *PIK3CA* that are common to colon cancer have been reported to be associated with resistance to cytotoxic agents or molecularly targeted drugs [46]. Inhibition of FLIP expression by siRNA has been demonstrated to induce death-ligand independent apoptosis in colorectal carcinoma cells [8] and has been shown to have supra-additive effects with TRAIL treatment in some colorectal cancer cells, although these cells were mostly TRAIL-sensitive [43,49]. Additionally, some chemotherapeutic agents have also been shown to inhibit FLIP expression and sensitize cells to treatment with TRAIL [41,47].

From our screen of 27 human colorectal cancer cell lines, we found that 14 of them responded to TRAIL, while the remaining 13 malignant cell lines were highly resistant up to a concentration of 250 ng/ml. In addition, two non-tumorigenic normal colon lines were also insensitive to TRAIL. Interestingly, we observed no correlation between TRAIL sensitivity and mutations of *KRAS*, *BRAF* or *PIK3CA* in our colorectal cancer cell panel. In addition, in a subset of this panel we were unable to find an association between TRAIL sensitivity and basal expression of TRAIL receptors or FLIP, suggesting that the mechanism of resistance for these cells was not at the level of death receptor expression or a known inhibitor of death receptor associated caspases. Intriguingly, we noted a transient apoptotic response to the ligand in a number of TRAIL-resistant colorectal cancer lines, similar to that reported in human prostate adenocarcinoma cells [17]. The mechanism of resistance remains unclear in colorectal cell lines where this effect is seen. We hypothesized that the development of resistance to TRAIL-induced apoptosis in these colorectal cancer cells may be due either to increased activity/expression of survival proteins or decreased activity/expression of pro-apoptotic factors.

PI3 Kinase and HSP90-client regulated pathways are often activated in colorectal cancer [20-22]. Targeting PI3 Kinase or HSP90 with potent selective inhibitors results in cancer cell growth inhibition and in some cases limited apoptosis [21-27]. Inhibitors of these molecular targets are progressing in Phase I/II clinical trials with evidence of therapeutic activity at well tolerated doses [22-24]. We have shown here that PI-103 and 17-AAG, as representative potent and selective PI3 Kinase/mTOR and HSP90 inhibitors, respectively, were able to increase sensitivity to TRAIL in colorectal cancer cells. In addition, in HT29 tumor cells that are resistant to PI-103 through rapid glucuronidation to an inactive metabolite [34], we showed that the metabolically stable, potent and selective pan-class I PI3 Kinase inhibitor and investigational clinical agent GDC-0941 [42,43] also increased sensitivity to TRAIL.

Our results suggest a mechanism by which PI-103 and 17-AAG increased sensitivity of colorectal cancer cells to TRAIL involving amplification of the TRAIL apoptotic signal through the decreased expression/activation of key pro-survival proteins on the PI3 Kinase/AKT axis and IKK/NFκ-B/IAPs pathway. Inhibition of AKT and IKK activation likely contributed to the observed decrease in expression of XIAP, possibly via inhibition of NFκ-B. Further experiments with isogenic overexpression or knock-out models will be required to definitively establish the mechanistic contribution of these pathways and their inhibitors to TRAIL resistance and the synergistic response to TRAIL treatment respectively. However, these studies were outside the scope of the present translational study that was focused primarily on exploring the therapeutic potential of combining TRAIL with HSP90 or PI3K inhibitors for the treatment of TRAIL-resistant tumors and providing pharmacodynamic biomarkers that support the combination and could be used clinically in a combination trial. Of interest, a previous study demonstrated that NO-Cbl, an analogue of vitamin B12 that delivers nitric oxide, when used in combination with TRAIL or other chemotherapeutic agents, caused increased apoptosis via decreased activity of AKT and IKK, resulting in reduced NFk-B activation and thus lower XIAP expression [48]. Similarly, bortezomib, an inhibitor of the proteasome, potentiates the TRAIL apoptotic signal via multiple mechanisms, including AKT, NFk-B and XIAP downregulation [49]. We showed that silencing XIAP followed by TRAIL treatment of TRAIL-resistant RKO colorectal tumor cells mimicked the effect of 17-AAG or PI-103 plus TRAIL. This finding supports the role of decreased XIAP expression in sensitization to TRAIL, particularly in the case of the 17-AAG and TRAIL combination where XIAP levels were reduced to undetectable after 24h of co-treatment in TRAIL-resistant RKO cancer cells. These observations are consistent with the demonstration that DR5 antagonist antibody combined with BV6, a potent IAP inhibitor, synergistically inhibited tumor growth via amplification of apoptosis in various tumor cell lines, including colorectal cancer models [50].

Thus, of potential translational and clinical interest, we have demonstrated that treatment with either the exemplar PI3 Kinase inhibitor PI-103 or the exemplar HSP90 inhibitor 17-AAG is able to amplify TRAIL-induced apoptosis in colorectal cancer cells *in vitro*. Importantly, to our knowledge for the first time, we have demonstrated that combinatorial treatment with 17-AAG and TRAIL resulted in an improved response compared to the individual agents *in vivo* in two TRAIL-resistant human colorectal cancer xenograft models. We showed that molecular markers for apoptosis, such as DNA breaks assessed by TUNEL, cleaved PARP, cleaved caspase 8 and XIAP levels, were all modulated *in vivo* by the co-treatment and this biomarker modulation was consistent with the therapeutic effects seen. In addition, we demonstrated that HSP90 client proteins such as IKKα and especially ERBB2 were depleted in both 17-AAG and, to a greater extent, in 17-AAG plus TRAIL treatment groups. Furthermore, our observations of the absence of, or very weak, effects on survival signaling following the combinatorial treatments in non-transformed CO841 colon epithelial cells suggest the possibility that these combinations may be relatively less toxic to normal cells.

In summary, we have shown that the decreased expression or activation of key survival molecules following treatment with PI3 Kinase/mTOR inhibitor PI-103 or HSP90 inhibitor 17-AAG in combination with TRAIL was associated with enhanced apoptosis in TRAIL-resistant colorectal cancer cells. Our results suggest a mechanistic explanation for the TRAIL sensitization by the mechanism-based small molecule inhibitors through inhibition of IKK/NFκ-B/IAPs and decreased expression of XIAP that will require further experiments to be confirmed. Notwithstanding the definitive proof of mechanism, our findings support the use of these survival-related molecules as pharmacodynamic biomarkers for monitoring the sensitization effect. Furthermore, we provide *in vivo* proof of concept and supportive biomarker data for the therapeutic activity of the representative HSP90 inhibitor 17-AAG plus TRAIL combination in two TRAIL-resistant human colorectal cancer xenograft models. We conclude that inducing apoptosis by TRAIL in combination with HSP90 or PI3 Kinase/mTOR inhibitors may represent a promising potential therapeutic approach for clinical evaluation in colorectal cancer.

## MATERIALS AND METHODS

### COMPOUND SUPPLY

PI-103 was provided by Piramed Pharma and GDC-0941 was purchased from Selleck Chemicals. 17-AAG was purchased from ChemiTek. Human recombinant human specific TRAIL was kindly provided by Dr. Ladislav Andera (Laboratory of Cell Signaling and Apoptosis, Institute of Molecular Genetics, Academy of Sciences of the Czech Republic). Caspase 8 inhibitor Z-IETD-FMK and caspase 3 inhibitor Z-DEVD-FMK were purchased from R&D Systems.

### CELL CULTURE AND CELL GROWTH INHIBITION ASSAY

Cell lines were obtained from ATCC and were further authenticated in-house by SNP profiling at the time of the experiments. Mutation status was also confirmed by mass-spectrometry-based sequencing of common oncogenic mutations. All were cultured in DMEM (Sigma Aldrich) and supplemented with 10% FBS (PAA Laboratories). Cells were maintained at 37° C in a humidified incubator 5% CO_2_. For growth inhibition assays, cells were seeded into a 96 well plate and Sulphorhodamine B (SRB) assay was carried out as described [34]. For combination assays 17-AAG was added 5 h before TRAIL; PI-103 and GDC-0941 were added simultaneously with TRAIL. All the treatments were performed for a total time of 96 h; the median effect analysis was used for calculation of combination indices (CI) [51]. For RKO cells synergism was determined using the method previously described by Hucl and colleagues [52].

### FLOW CYTOMETRY

Apoptosis was measured by flow cytometry. Staining was carried out by suspending 1×10^5^ cells in 0.5 ml 1X Annexin V binding buffer plus FITC-Annexin V (Cambridge Bioscience) and propidium iodide (Invitrogen).

To examine cell surface TRAIL receptor expression, antibodies to DR4, DR5, DcR1 and DcR2 (Axxora) were used followed by secondary FITC-conjugated antibody (Axxora). Mouse IgG1 antibodies were used as an isotype control. Propidium iodide was added to each sample 5min before analyzing by flow cytometry.

### IMMUNOBLOTTING

Cells were lysed in lysis buffer (Cell Signaling) containing protease inhibitors (Roche). Immunoblotting was performed according to standard procedures [34]. All antibodies used were from Cell Signaling except for ERBB2 (Santa Cruz Biotechnologies) and GAPDH (Bio-Rad).

### siRNA TRANSFECTION

XIAP siRNAs were synthesized by Qiagen with the following target sequences: oligo 4 CACGTACTTGTGCGAATTATT and oligo 5 AAGTGCTTTCACTGTGGAGGA. FLIP siRNAs were synthesized by Qiagen: oligo 3 Hs_CFLAR_3 HP siRNA and oligo 4 Hs_CFLAR_4 HP siRNA. The All Star oligo (Qiagen) was used as negative control. Transfection of cells with 20 nM siRNAs was carried out using oligofectamine (Invitrogen) according to the manufacturer's protocol.

### EFFICACY AND PHARMACODYNAMIC STUDIES IN HUMAN TUMOR XENOGRAFTS

All experiments were performed in accordance with the local ethical review panel, the UK Home Office Animals Scientific Procedures Act, 1986 and UKCCCR and NCRI guidelines [53]. Human colorectal tumor xenografts were obtained using sub-cutaneous implantation of 5×10^6^ RKO or SW620 cells in Nude/nu mice (Harlan). Therapy (long-term study in the text) was initiated once tumors were established and continued for 15 days in the RKO model and 23 days in the SW620 model. Mice were dosed i.p. 5 days per week with 50 mg/Kg/day for RKO and 40 mg/Kg/day for SW620 17-AAG in the morning and with 15 mg/Kg/day TRAIL in the afternoon. For pharmacodynamic biomarker analysis mice bearing RKO tumor xenografts were treated for 4 days (short-term study in the text) as described above. Tumors were removed at 16 h after last TRAIL dose. Each tumor was divided into two parts, one half being formalin-fixed and the other half frozen, for analysis by immunohistochemistry (IHC) and immunoblot respectively.

### IMMUNOHISTOCHEMISTRY: TUNEL ASSAY

Apoptosis was measured in paraffin-embedded tumor sections using the In Situ Cell Death Detection Kit (POD, Roche) following the manufacturer's instructions. Nuclei were counterstained with haematoxylin. Analysis of the nuclear staining was performed with Aperio software, intensity of the staining was in a range from 0 (no staining) to 3+ (strong staining), only 3+ and 2+ positive nuclei were considered for final analysis.

### STATISTICAL ANALYSIS

Data presented were analyzed by unpaired Student's t-test; p values less than 0.05 were accepted as statistically significant different compared to controls.

## Supplementary Figures


