# Supplementary material for: Combining TRAIL with PI3 Kinase or HSP90 inhibitors enhances apoptosis in colorectal cancer cells via suppression of survival signaling
Source: Oncotarget. 2013 Jul 14;4(8):1185–98. doi: 10.18632/oncotarget.1162 (PMC3787150; doi:10.18632/oncotarget.1162)
Supplement: Supplementary file 1 [file oncotarget-04-1185-s001.pdf]

Combining TRAIL with PI3 kinase or HSP90 inhibitors enhances apoptosis in colorectal cancer cells via suppression of survival signaling - Saturno et al

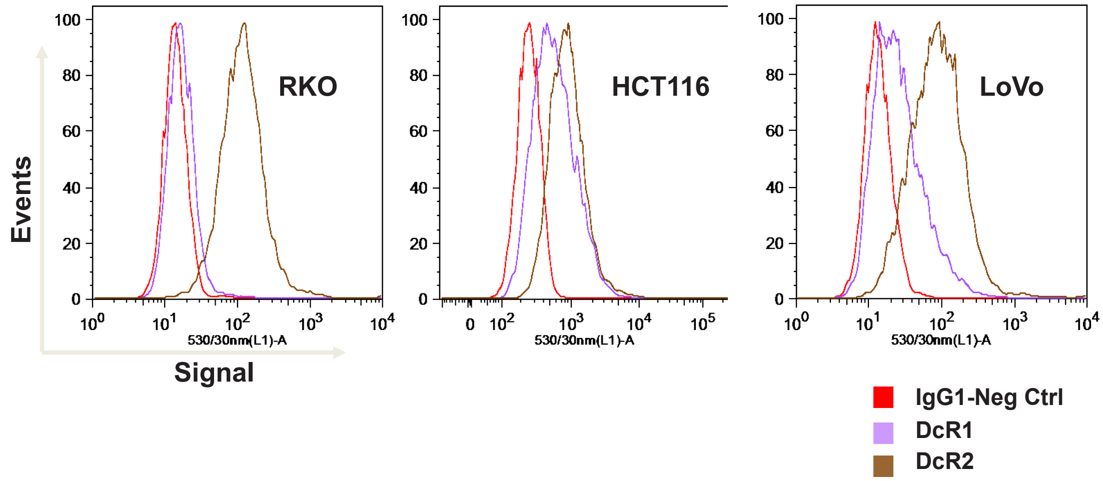

Supplementary Figure 1: Expression analysis of TRAIL decoy receptor DcR1 and DcR2 by flow cytometry. Histograms represent the IgG1 negative control (red), DcR1 (purple) and DcR2 (brown) on the surface of live cell populations. Cells were counterstained with propidium iodide and PI-positive dead cells were excluded from analysis. The intensity of the fluorescent signal is reported on the X axis. Plots are representative of three independent experiments.

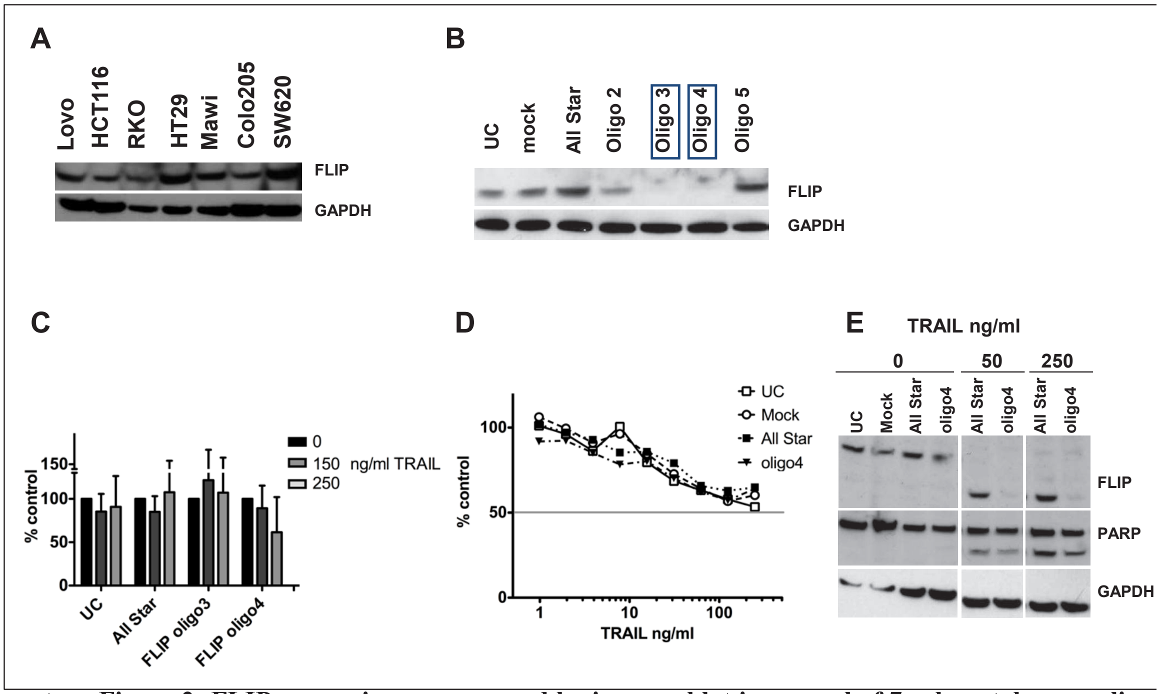

Supplementary Figure 2: FLIP expression was assessed by immunoblot in a panel of 7 colorectal cancer lines (panel A), no correlation with TRAIL resistance and FLIP levels was observed. Panel B: RKO cells were transfected with two different siRNA oligos targeting FLIP. UC = untreated control, Mock = oligofectamine only, All Star = oligo control, oligo 2, 3, 4 and 5 = four different oligos targeting FLIP. Oligo 3 and 4 were chosen for further analysis. Cell number was measured using SRB (panel C) after FLIP silencing (48 h) followed by TRAIL 150 ng/ml or 250 ng/ml treatment for additional 24 h (left). All the treatments are shown as percentage of untreated cells. Relative to All Star control, FLIP Oligo 3 and Oligo 4 did not significantly decrease viability after TRAIL treatment at either TRAIL concentration. Panel D: cells were treated with siRNA for 48 h and then exposed to different concentrations of TRAIL for 96 h. No difference was observed between All Star negative control oligo and FLIP oligo 4 transfected cells (data representative of two independent experiments). Panel E: FLIP and PARP expression (total and cleaved) in RKO cells transfected with FLIP Oligo 4 (48 h) and controls with or without TRAIL treatment at two different concentrations (50 and 250 ng/ml for additional 24 h) were measured by immunoblotting; GAPDH was used as loading control. No changes in PARP cleavage were observed in FLIP siRNA cells compared to the All Star negative control transfected cells after TRAIL treatment.

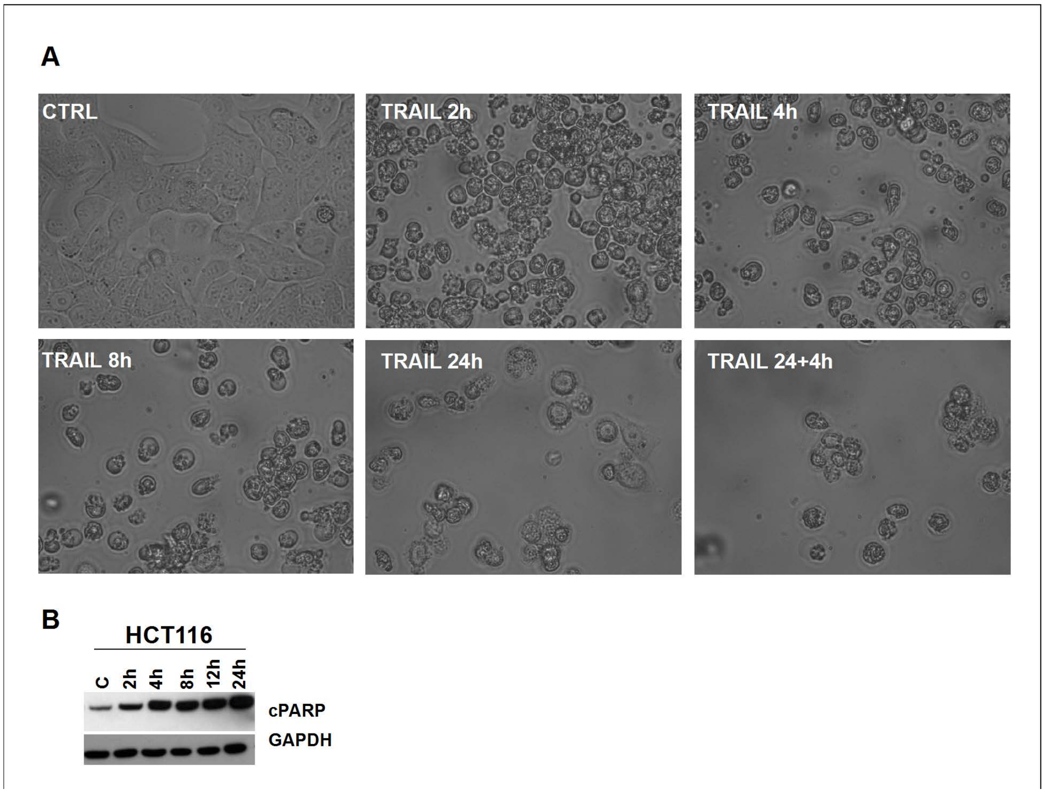

Supplementary Figure 3: TRAIL time course in HCT116 cells. Panel A: images of HCT116 cells at time 0 or treated with TRAIL 5 ng/ml for 2, 4, 8, 24 h or 24 h plus additional 4 h were taken using an inverted light microscope (Leica), 20X magnification. TRAIL treatment induced the rounded and small appearance of apoptotic cells at all time of treatment. Cleavage of PARP was assessed by immunoblot at time 0 (C), 2, 4, 8, 12 and 24 200 ng/ml TRAIL treatment (panel B).

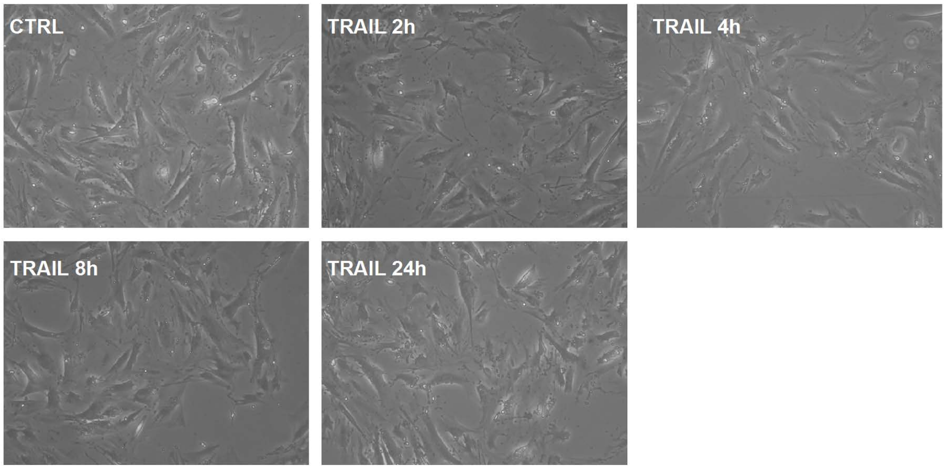

Supplementary Figure 4: TRAIL time course in CO841 cells. Images of CO841 cells at time 0 or treated with TRAIL 200 ng/ml for 2, 4, 8, 24 h were taken using an inverted light microscope (Leica), 10X magnification.

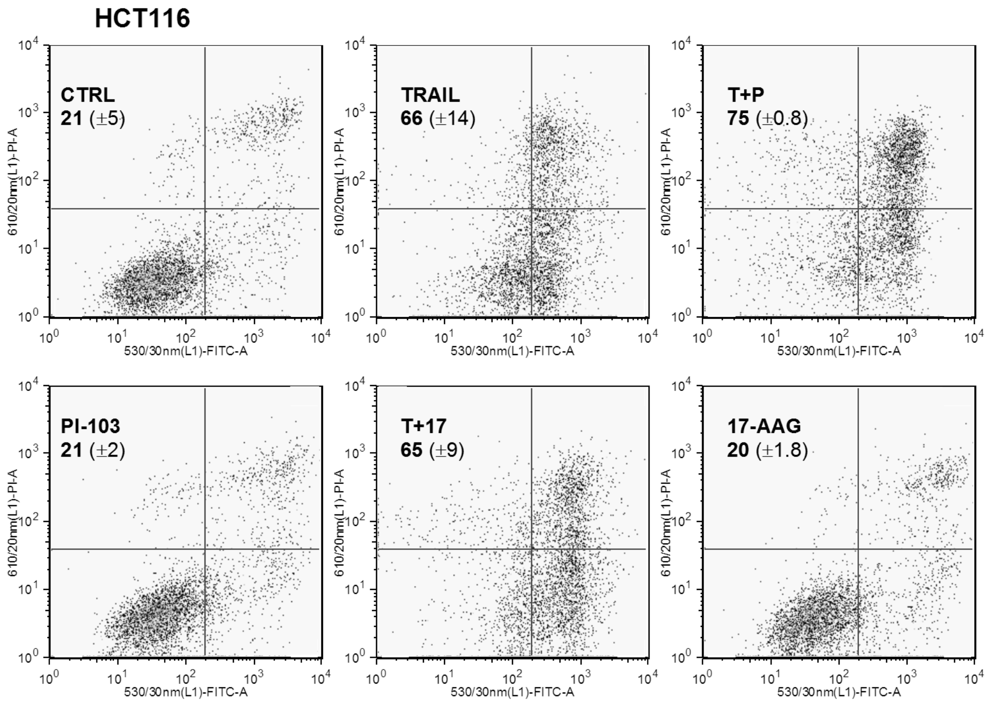

Supplementary Figure 5: Apoptosis was measured in HCT116 colorectal cancer cells by flow cytometry using FITC-Annexin V and PI staining. Percentage means (± s.d.) of FITC-Annexin V plus FITC/PI stained cells are reported for each treatment. Plots are representative of three independent experiments.

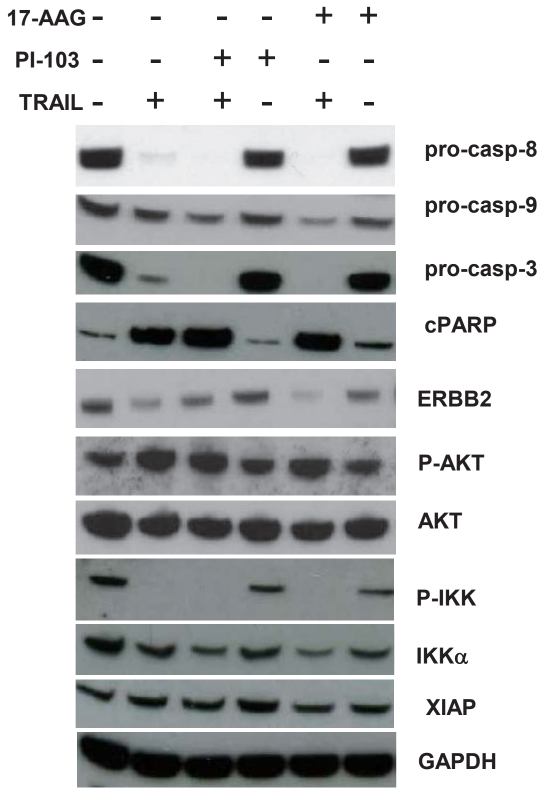

Supplementary Figure 6: Effect of combination treatments with TRAIL (12.5 ng/ml) plus PI-103 (2.5 □icroM) or 17-AAG (117.5 nM) in TRAIL-sensitive HCT116 cells. Immunoblots for pro-caspase 8, 9, 3 levels and PARP cleavage after single or combination treatments are shown. A decrease in pro-caspase levels indicates greater caspase cleavage. An increase in cleaved PARP levels indicates greater apoptosis. Immunoblot for ERBB2, AKT and AKT[Ser473], IKK□□□IKK□[Ser176], c-IAP1 and XIAP are shown.

A

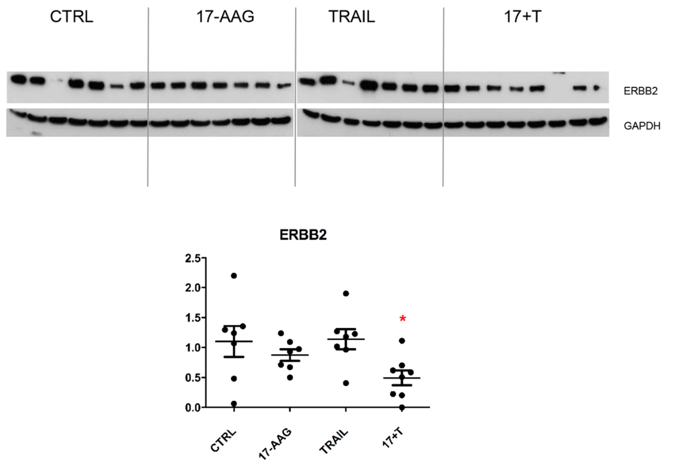

B

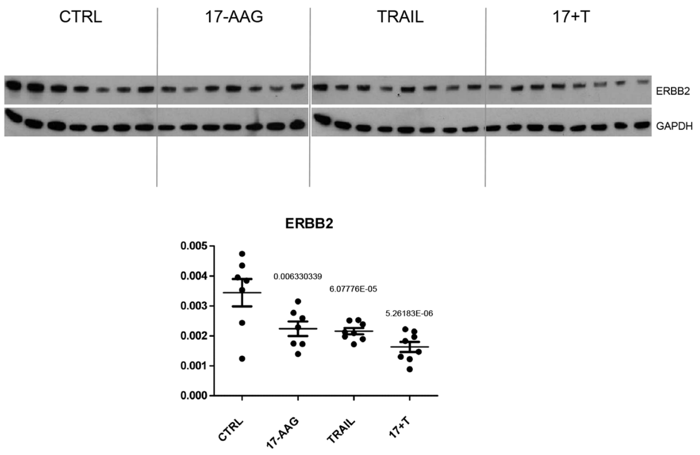

Supplementary Figure 7: Immunoblot analysis for ERBB2 as an efficacy biomarker at the end of therapy (long-term study). Panel A: SW620 colorectal tumor xenograft model, each lane corresponding to one representative tumor/animal, (*) denotes p≤0.05. Panel B: RKO colorectal tumor xenograft model, each lane corresponding to one representative tumor/animal, numbers represent p values.
